# The 3N Model and collective support for extreme measures to combat COVID-19

**DOI:** 10.1371/journal.pone.0335241

**Published:** 2025-11-04

**Authors:** Mirra Noor Milla, Jocelyn J. Bélanger, Winnifred R. Louis, Haykal Hafizul Arifin, Umar H. Sulaiman, Aly Lamuri, Norberta Fauko Firdiani

**Affiliations:** 1 Faculty of Psychology, Universitas Indonesia, Depok, West Java, Indonesia; 2 Arts and Sciences, Carnegie Mellon University in Qatar, Doha, Qatar; 3 School of Psychology, University of Queensland, Brisbane, Australia; 4 Faculty of Medicine, Universitas Indonesia, Depok, West Java, Indonesia; 5 Faculty of Psychology, Universitas Diponegoro, Semarang, Central Java, Indonesia; PLoS ONE, UNITED STATES OF AMERICA

## Abstract

Understanding resistance to COVID-19 measures is crucial, since it undermines public health efforts during crises. Building on prior research showing the crucial role of individual psychological factors in shaping responses to such efforts, we focus on psychological factors involved. Specifically, we examine such psychological factors that shape support for COVID-19 measures by conceptualizing them as extreme measures—restrictions that significantly alter daily life—thus enabling the application of the 3N model of extreme behavior. Drawing on data from the multinational dataset (N = 62,983) across 114 countries, we tested the role of two types of needs/losses: collective loss and personal loss. The results reveal a differential pattern: collective loss is associated with lower support for extreme measures, while personal loss is associated with higher support. Both effects are mediated by perceived social network norms, though the strength of mediation differs. These findings extend the 3N model by highlighting how distinct types of loss shape responses to extreme measures, and they offer implications for designing public policies that address both individual and collective concerns.

## Introduction

The global COVID-19 outbreak has caused a significant number of deaths worldwide. Mitigation strategies often disrupt the daily lives of citizens and constrain rights and privileges. The enforcement of regulations aimed at curbing the spread of COVID-19 has triggered social unrest in various countries, leading to peaceful protests and violent confrontations with law enforcement [1]. Despite vaccination’s proven necessity and effectiveness in reducing COVID-19 transmission [2], phenomena such as vaccine hesitancy are still an issue in the context of public health policy and disease prevention [3]. Thus, it is critical to comprehend the factors that may account for resistance to anti-COVID policies.

We propose that a thorough investigation of socio-cognitive factors is crucial for comprehensively understanding the mechanisms at work in this context. Gajda et al. [4] found that personal characteristics significantly influence the intention of radical anti-vaccine collective action, particularly when social norms support non-compliance. This underscores the importance of understanding how individual and collective needs and narratives shape attitudes toward COVID-19 measures. Spiliopoulos [5] showed how early high uncertainty during the COVID-19 pandemic justified stricter measures, but as uncertainty decreased, people’s ability to judge the risk of spread improved, leading to more moderate responses. Thus, developing a model that comprehensively examines the psychological factors that may account for resistance towards COVID-19 measures is essential to advance understanding and improve public health strategies for addressing it.

In this study, we define and operationalize extreme measures as restrictive public health policies—specifically mandatory vaccination, quarantine of exposed individuals, and the reporting of suspected cases—that were deployed worldwide during the COVID-19 pandemic to contain transmission. While these interventions draw from standard public health toolkits, we label them extreme to capture their unprecedented scale, degree of intrusiveness, and enforcement during COVID-19, distinguishing them from responses in earlier pandemics through comparative data. By early April 2020, more than half of the world’s population—approximately 3.9 billion people—were under some form of lockdown in over 90 countries or territories [6]. Data from the Oxford COVID-19 Government Response Tracker show that many governments adopted combinations of policies including closure of non-essential businesses, stay-at-home orders, and digital tracking [7]. The economic consequences were also substantial: global GDP contracted sharply, with emerging and developing economies experiencing severe disruptions to trade, employment, and supply chains ( [8,9].

Comparatively, during earlier pandemics—such as the 1918 influenza—interventions were indeed implemented, but their form and scope differed significantly from those seen during COVID-19. Nonpharmaceutical interventions such as school closures, bans on gatherings, and quarantines were widely used in U.S. cities, but their timing and duration varied considerably [10]. More broadly, historical accounts emphasize that before the 20th century, the capacity for large-scale, internationally coordinated enforcement was extremely limited; measures were largely local or regional, reflecting the political and technological constraints of the time [11]. Prior research shows that individuals often perceive such policies as extreme. Ramirez & Wood [12] demonstrates that restrictive COVID-19 policies can be construed as threats to the social order, prompting individuals with high authoritarian dispositions—regardless of political orientation—to resist these policies and align with anti-COVID groups. We therefore argue that resistance should be analyzed within a threat-to-social-order framework, in which COVID-19 policies are categorized as extreme measures [see also 5]. In sum, we define “extreme” COVID-19 measures specifically as restrictive policies.

Conceptualizing COVID-19 management policies as extreme measures invites the application of the 3N Model (needs, networks, narratives) derived from the significance quest theory [13]. Kruglanski et al. [13] proposed the 3N Model of radicalization as a framework for understanding extreme beliefs and actions. This model includes three key components: Need, Narrative, and Network. Need refers to the drive for significance, which is the fundamental desire to achieve recognition, value, and a sense of worth. This need can become more pronounced due to experiences like personal failure, rejection, or social alienation. Individuals who feel a lack of significance may become more susceptible to radicalization as they seek ways to restore their sense of worth and identity. The “needs” component is highly salient during the pandemic, where widespread uncertainty, economic disruption, social isolation, and mortality salience undermine individuals’ sense of personal significance, thereby creating motivational deficits. Research on COVID-19 has shown that these stressors intensify the quest for significance, motivating individuals to seek behaviors that restore affirmation [14]. Amidst a pandemic, individuals may encounter adverse experiences that they attribute to unfair treatment by others or authorities. Various measures have assessed the effects of these experiences, including mental health concerns, stress, and adverse outcomes like financial difficulties and restrictions on activities, impacting individuals and their close relationships [15–18]. These negative experiences, both personal and collective, can activate a quest for significance [19]. These experiences can induce feelings of entitlement, resulting in a desire to advocate for their rights, including policies regarding COVID-19 management.

Pandemic-induced needs alone cannot fully explain why some individuals adopt extreme measures to regain significance while others reject them. To address this puzzle, the 3N Model highlights the roles of networks and narratives. Networks provide the social context that validates specific narratives, while narratives embedded within these networks offer pathways to restore significance. Narrative involves the ideologies and belief systems that provide a framework for interpreting experiences and justifying actions. These ideologies often rationalize extreme behaviors by presenting them as necessary or morally justified responses to perceived injustices or threats. Narratives help individuals make sense of their experiences and can motivate the adoption of radical beliefs or engagement in violent actions. Networks encompass the social connections and groups that reinforce and support radical ideologies. These networks offer a sense of belonging and validation for individuals who feel isolated or disenfranchised. They also play a crucial role in spreading and normalizing radical ideas and mobilizing individuals toward extreme actions. Social networks provide both emotional support and practical resources that facilitate the persistence of radical beliefs. For some individuals, their networks supply alternative narratives—such as conspiracy theories [e.g., 20]. In short, the 3N Model functions as a versatile lens for analyzing how crises like the pandemic motivate policy-related behaviors, offering insights into the dynamics of both support and opposition.

How the 3N model works might not be as straightforward as the previous exposition described. One overlooked aspect is how the need component of the model interacts with the other two components. Decades of psychological research have established the motivated nature of human [21–24]. Individuals could be selective in what they attend to and might be biased in accordance with their needs and motives. The perception of descriptive norms—that is, beliefs about what others in one’s social network typically do—may be influenced by individuals’ already present needs and motives [25]. Previous research has described that there is a difference between the source of individuals’ needs for significance [19,26]. The triggering significance loss can be due to individual or personal humiliation (such as financial constraints, loneliness, or shame), or threats to a collective or group can also be the source of such loss. Individuals who identify with a group may then feel a need for significance when their social groups are humiliated. Thus, significance loss can arise not only from personal humiliation but also from collective degradation, with such needs for significance shaping how individuals perceive social norms within their networks and, consequently, orient themselves toward extreme narratives.

Previous work of Jasko et al. [19] explain how both types of loss could lead individuals to the same extreme behavior. This study advances the 3N Model by examining how distinct sources of significance loss—personal versus collective—activate different needs that shape perceptions of social norms and, in turn, support for extreme pandemic measures. We argue that personal losses orient individuals toward self-focused needs, making them perceive norms as supportive yet less decisive in their stance, which leads them to endorse restrictive policies because they directly address their safety concerns. Conversely, collective losses activate group-focused needs, heightening sensitivity to group-based restrictions, which fosters perceptions of rejection norms and ultimately opposition to such policies. By testing these mechanisms in the underexplored context of a global pandemic, this study contributes to a more nuanced understanding of how significance loss channels normative perception and motivates support for extreme social measures.

### Current study

The present study examines the importance of significance in the context of both personal and collective loss. As discussed in the previous section, prior research has made distinction on the loss experienced by individuals to understand how narratives from violent extremist support groups play a role. Jasko et al. [19] demonstrated that when individuals perceive collective grievances—whether material or symbolic—as significant, they are more likely to support extreme and violent political strategies. We further extend this distinction by testing whether this different type of loss result in different type of response support towards extreme measure against COVID-19. Specifically, we propose two hypotheses:

1. Individuals who experience personal loss of significance are more likely to support extreme measures to combat COVID-19 mediated by social network restrictive norm;
2. Individuals who experience collective loss of significance are less likely to support extreme measures to combat COVID-19 mediated by social network restrictive norm.

We utilized aggregate data from different countries, assuming that the 3N Model explains behavior at the individual level. In our approach, differences in national COVID-19 policies are assumed to be incorporated into individuals’ perceptions of prevailing norms and narratives.

## Methods

### Source data and respondent characteristics

The present study utilized data from the PsyCorona Study dataset, which is a multinational initiative focused on examining psychological and behavioral responses to the Covid-19 pandemic. This survey received ethical approval from the University of Groningen’s psychology ethics committee (PSY-1920-S-0390) and New York University Abu Dhabi (HRPP-2020–42). Informed consent was obtained from all participants prior to survey completion. Details and quality control procedures are provided in the CHERRIES checklist [27] in the Supplementary File.

Leander et al. [28] provide an overview of the data, while the PsyCorona dataset is detailed by Agostini et al. [29]. For this study, we analyzed complete-case baseline data of the relevant non-demographic variables from 62,983 participants (original sample size: N = 64,381). Of these, 61.4% were female, 38.1% were male, 0.5% identified as other genders, and 144 participants without gender data. The measures were translated into 30 languages, and participants were from 114 countries. The translations were necessary due to the diverse geographic representation of the sample. We translated all initial measurement tools from English into the languages of each participating country and conducted expert evaluations to ensure the equivalence of these translations. The study employed longitudinal data, inviting participants to engage in a follow-up multi-level study after completing the baseline survey. However, due to a substantial drop-off rate of over 90% from the baseline to later waves, the analysis did not include data from subsequent waves.

The PsyCorona Dataset was extensive and encompassed a wide range of psychological factors at the individual level. Nevertheless, this study specifically examined collective support for extreme measures and the three characteristics known as 3N (needs, narratives, and networks). The respondents were categorized into different age groups, namely 18–24 years (23.5%), 25–34 years (24.4%), 35–44 years (19.1%), 45–54 years (14.3%), 55–64 years (10.9%), 66–75 years (6.7%), 75–85 years (0.9%), over 85 years (0.1%), and 176 participants with no age data. The participants’ educational attainment was as follows: 30.8% held a bachelor’s degree, 23.3% had completed higher education, 16.7% had obtained a master’s degree, 13.0% had completed general secondary education, 9.6% had vocational education, 5.2% held a PhD degree, 1.4% had completed primary education, and 257 participants do not have educational attainment data.

### Measures

**Loss of Personal Significance** (Personal) was a latent variable measured by three indicators drawn from the literature, namely loneliness [30], perceived financial strain [13] and happiness (reverse-scored) which refers to negative emotional states [26,31].

Loneliness can be a significant factor contributing to a loss of personal significance. When individuals feel socially isolated and disconnected from others, they may experience a diminished sense of self-worth and importance. Loneliness was measured using a 3-item 5-point scale evaluating perceived loneliness during the past week [32]: “Did you feel lonely?” “Did you feel isolated from others?” “Did you feel left out?” (−2 = Never, −1 = Rarely, 0 = Sometimes, 1 = Often, 2 = All the time).

Perceived financial strain can also impact one’s sense of personal significance. Financial difficulties and financial stress can lead to feelings of helplessness and a reduced sense of control over one’s life. Perceived financial strain is measured using a 3-item 5-point scale to assess financial-related issues [33]: “I am financially strained,” “I often think about my current financial situation,” and “Due to my financial situation, I have difficulties paying for my expenses” (−2 = Strongly disagree, −1 = Disagree, 0 = Neither agree nor disagree, 1 = Agree, 2 = Strongly agree).

Happiness is closely linked to an individual’s well-being and personal satisfaction. A lack of happiness or a low score on a happiness scale may indicate a diminished sense of personal significance. A single-item measure devised by Abdel-Khalek [34] was employed: “In general, how happy would you say you are?” (1 = Extremely unhappy, 10 = Extremely happy).

**Loss of Collective Significance** (Collective) was a latent variable with 2 items [35]. Collective loss of significance describes a situation when group members believe that their needs and interests are consistently ignored or that their group is disadvantaged compared to others. It can lead to frustration, discontent, and a desire to address these issues collectively. The items are as follows: “Not a lot is done for people like me in this country” and “If I compare people like me against other people in this country, my group is worse off” (−2 = Strongly disagree, −1 = Disagree, 0 = Neither agree nor disagree, 1 = Agree, 2 = Strongly agree).

**Descriptive Norms** (Norm) COVID-19 Perceived Community Response (norm) is a latent variable of 2 items used to measure community response towards COVID-19. We adapted the measurement for belief of social norms and conformity from Leander et.al. [36], carefully tailoring it to reflect the unique challenges and circumstances of the COVID-19. The measurement captures the actions taken by a part or the whole of a community. The items are: (1) “Right now, people in my area do self-isolate and engage in social distancing” (−3 = Strongly disagree, −2 = Disagree, −1 = Somewhat disagree, 0 = Neither agree nor disagree, 1 = Somewhat agree, 2 = Agree, 3 = Strongly agree), (2) “To what extent is your community developing strict rules in response to the Coronavirus?” (1 = Not at all, 6 = Very much).

**Collective support for extreme measures to combat COVID-19** (Extreme) is a latent variable consisting of 3 items to measure collective actions amidst the COVID-19 pandemic [37]. Respondents are asked to respond to the statement “I would sign a petition that supports...” (1) mandatory vaccination once a vaccine has been developed for Coronavirus, (2) mandatory quarantine for those that have Coronavirus and those that have been exposed to the virus (3) reporting people who are suspected of having Coronavirus” (−3 = Strongly disagree, −2 = Disagree, −1 = Somewhat disagree, 0 = Neither agree nor disagree, 1 = Somewhat agree, 2 = Agree, 3 = Strongly agree).

### Research design and data analyses

We utilized the 3N Model to explore the relationships between needs, networks, and narratives in the context of the COVID-19 pandemic. We distinguished between the need for personal and collective significance, measuring collective significance loss through disempowerment and personal significance loss through perceived financial stress, loneliness, and happiness. A structural equation model with a Diagonally Weighted Least Square (DWLS) estimator was employed because ordinal data [38] were fitted to test the hypotheses. The treatment of the missing data was listwise deletion. Thresholds between the ordinal responses were freely estimated. We also note that the model’s latent variables were defined with two indicators each [39]. As Steiger [40] cautioned, models with only two indicators per factor rely heavily on identification constraints (e.g., fixed loadings), which can interact with other model constraints and affect parameter interpretation and model fit. The lack of redundancy limits the assessment of unidimensionality and may reduce estimate stability. Thus, in interpreting the effects of Collective Need and Norms on Extreme support, caution is warranted. The analysis was first conducted by JASP software; however, as the software wasn’t able to provide threshold information, the analysis was conducted using lavaan 0.6–19 [41]. The difference did not alter the conclusions.

We fitted a latent mediation model where personal and collective loss are configured to predict restrictive norms, which predict collective support for extreme measures to combat COVID-19. Considering the large sample size of the data analyzed, chi-square statistics will be too sensitive to detect errors in the model. We utilized multiple fit indices to evaluate the fitness of the model. The recommended cutoff value for CFI and TLI indices is.95, while for RMSEA is.06 and SRMR is.08 [42]. We made modifications to the model as needed. Observed variables were standardized before estimation.

## Results

### Model Fit and Factor Loading

Table 1 provides the dsecriptive statistic of the observed indicators while Table 2 provides the correlation matrix for the observed measures used in the model. We made no modifications in the measurement model. The resulting model demonstrated excellent fit to the data, χ²(69) = 9672.96, *p* < .001, RMSEA = .047, SRMR = .038, CFI = .993, TLI = .990. TLI and CFI were considered to fit well [43], and so was the RMSEA [44]. The model specified six latent variables: *Lonely*, *Financial*, and *Personal Need; Collective Need*, *Restrictive Norms, and Extreme support*. The *Personal Need* construct was defined as a second-order factor influenced by *Lonely* and *Financial*. The *Extreme* outcome variable was regressed on *Collective Need*, *Personal Need*, and *Restrive Norms*, while *Norms* were predicted by both *Personal* and *Collective Need*.

**Table 1:** Descriptive Statistic (Unstandardized).

Variable	n	mean	sd	median	min	max	range	skew	kurtosis
lone01	62983	2.42	1.15	2	1	5	4	0.35	−0.77
lone02	62983	2.67	1.23	3	1	5	4	0.17	−0.99
lone03	62983	2.08	1.15	2	1	5	4	0.81	−0.29
happy	62983	6.33	2.02	7	1	10	9	−0.44	−0.09
fail01	62983	−0.05	1.15	0	−2	2	4	0.03	−0.81
fail02	62983	−0.40	1.11	−1	−2	2	4	0.32	−0.65
PFS01	62983	−0.02	1.21	0	−2	2	4	−0.02	−0.98
PFS02	62983	0.58	1.13	1	−2	2	4	−0.70	−0.26
PFS03	62983	−0.24	1.24	0	−2	2	4	0.21	−1.01
c19RCA01	62983	1.27	1.84	2	−3	3	6	−0.96	−0.11
c19RCA02	62983	2.07	1.32	3	−3	3	6	−1.81	3.27
c19RCA03	62983	1.17	1.80	2	−3	3	6	−0.84	−0.31
c19NormDo	62983	1.30	1.51	2	−3	3	6	−0.94	0.32
c19IsStrict	62983	4.12	1.40	4	1	6	5	−0.44	−0.57

**Table 2:** Correlations between observed variables.

	1	2	3	4	5	6	7	8	9	10	11	12	13
lone01	–												
lone02	0.62***	–											
lone03	0.62***	0.58***	–										
happy	−0.41***	−0.33***	−0.38***	–									
fail01	0.18***	0.17***	0.2***	−0.2***	–								
fail02	0.19***	0.18***	0.23***	−0.2***	0.49***	–							
PFS01	0.24***	0.21***	0.26***	−0.25***	0.33***	0.37***	–						
PFS02	0.21***	0.2***	0.21***	−0.19***	0.27***	0.26***	0.6***	–					
PFS03	0.24***	0.21***	0.26***	−0.24***	0.34***	0.38***	0.78***	0.55***	–				
c19RCA01	0.05***	0.03***	0.01	0.01	−0.02***	0	−0.01	0.05***	0	–			
c19RCA02	0.02***	0.04***	−0.04***	0.03***	0.01**	−0.02***	0.02***	0.1***	0.01***	0.47***	–		
c19RCA03	0.06***	0.03***	0.03***	0.01***	0.05***	0.07***	0.09***	0.1***	0.12***	0.39***	0.53***	–	
c19NormDo	−0.05***	−0.01**	−0.06***	0.12***	−0.1***	−0.06***	−0.03***	0.01	−0.03***	0.15***	0.17***	0.11***	–
c19IsStrict	−0.06***	−0.03***	−0.05***	0.14***	−0.14***	−0.07***	−0.04***	−0.02***	−0.03***	0.07***	0.1***	0.09***	0.34***

*p < .05, **p < .01, ***p < .001

### Regression

**Fig 1:**
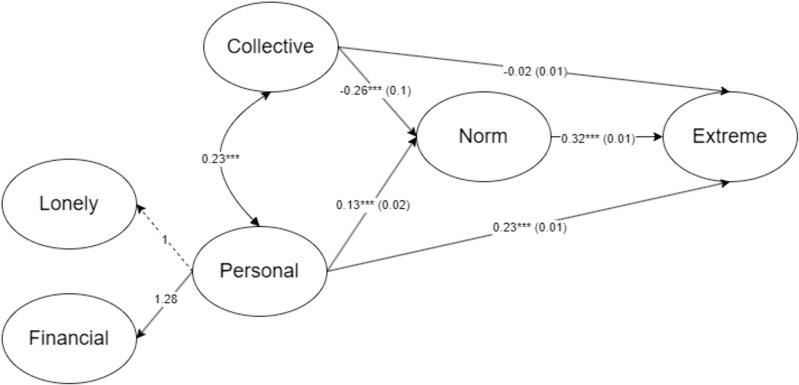
Needs-Narrative-Network Model of Collective Support Extreme Measure to Combat COVID-19 (Only the latent score is shown). ***p < .001. Standard errors are inside parentheses.

As seen in Fig 1, both loss of personal and collective significance covary with one another (b = .23 95% CI [.22,.24]). However, loss of collective significance, as reflected by disempowerment, has negative association towards social networks’ restrictive norms, *b* = −0.26, 95% CI [−0.28, −0.23]. On the other hand, after controlling for collective loss, the loss of personal significance is associated positively with social networks’ restrictive norms, *b* = 0.13, 95% CI [0.09, 0.16].These findings implied that perception of being disempowered collectively was associated with lower social network norms of restrictive measures. In contrast, personal experiences of loss of significance were associated positively with social network norms supporting restrictive measures. Social network norms then determined collective support for the extreme measures, *b* = 0.32, 95% CI [0.31, 0.34].

Analysis of the indirect effects (Table 3) determined that loss of personal significance positively predicted collective support for extreme measures through social network norms, *b* = 0.04, 95% CI [0.03, 0.05]. In contrast, loss of collective significance negatively predicted collective support through social network norms, *b* = −.08, 95% CI [−0.09, −0.08]. Analysis of the direct effects (Table 3) shows that loss of personal significance (b = 0.23, 95% CI [0.21, 0.26]), but not collective significance (b = −0.02, 95% CI [−0.03, 0]), positively predicted significantly extreme measures. This finding implies social network norms mediate more the relationships between loss and extreme measures, with a more pronounced effect for the losses that are collective in nature. The rest of the information regarding factors loading is present in Table 4.

**Table 3:** Direct effects & Indirect Effects.

Effect	b	β	SE	z	p	95% CI
Collective → Extreme	−0.02	−0.02	0.01	−1.69	0.24	[-0.03, 0]
Norm → Extreme	0.32	0.35	0.01	51.16	<.001	[0.31, 0.34]
Personal → Extreme	0.23	0.16	0.01	17.37	<.001	[0.21, 0.26]
Collective → Norm	−0.26	−0.26	0.01	−21.05	<.001	[-0.28, -0.23]
Personal → Norm	0.13	0.08	0.02	6.82	<.001	[0.09, 0.16]
Collective → Norm → Extreme	−0.08	−0.02	<0.01	−20.13	<.001	[-0.09, -0.08]
Personal → Norm → Extreme	0.04	0.35	0.01	6.8	<.001	[0.03, 0.05]

**Table 4:** Items and Factor Loading.

Latent	Code	Items	b	β	SE	z	p	95% CI
Lonely	lone01	Did you feel lonely?	1	0.85	–	–	–	–
Lonely	lone02	Did you feel isolated from others?	0.92	0.78	< 0.01	244.78	<.001	[0.91, 0.93]
Lonely	lone03	Did you feel left out?	0.98	0.83	< 0.01	246.96	<.001	[0.97, 0.98]
Lonely	happy	In general, how happy would you say you are?	−0.63	−0.53	< 0.01	−205.48	<.001	[-0.64, -0.62]
Collective	fail01	Not a lot is done for people like me in this country	1	0.71	< 0.01	–	–	–
Collective	fail02	If I compare people like me against other people in this country, my group is worse off	1.06	0.76	0.01	152.72	<.001	[1.05, 1.08]
Financial	PFS02	Due to my financial situation, I have difficulties paying for my expenses	1	0.72	–	–	–	–
Financial	PFS03	I often think about my current financial situation,	1.3	0.93	< 0.01	284.6	<.001	[1.29, 1.31]
Financial	PFS01	I am financially strained,	1.25	0.9	< 0.01	310.55	<.001	[1.25, 1.26]
Extreme	c19RCA01	I would sign a petition that supports	1	0.65	–	–	–	–
Extreme	c19RCA02	I would sign a petition that supports	1.36	0.88	0.01	134.64	<.001	[1.34, 1.38]
Extreme	c19RCA03	I would sign a petition that supports	1.13	0.73	0.01	154.46	<.001	[1.12, 1.15]
Norm	c19NormDo	Right now, people in my area do self-isolate and engage in social distancing	1	0.69	–	–	–	–
Norm	c19IsStrict	To what extent is your community developing strict rules in response to the Coronavirus?	0.77	0.54	0.01	52.99	<.001	[0.74, 0.8]
Personal	Lonely	2nd order factor	1	0.51	–	–	–	–
Personal	Financial	2nd order factor	1.28	0.77	0.01	123.29	<.001	[1.26, 1.3]

## Discussion

To investigate how personal and collective loss experiences during the pandemic influence support for extreme measures, we apply the 3N Model to distinguish between these types of loss. We assessed the likelihood of collective support for extreme COVID-19 measures. Using structural equation modelling, we show that the need for significance is associated with support for extreme measures against COVID-19, mediated by social network norms. Individuals experiencing personal loss are more likely to view their social networks’ norms as restrictive and thus advocate for stringent measures against COVID-19. Conversely, those facing significant collective loss are inclined to view social network norms as less restrictive, reducing their likelihood of supporting extreme measures.

In alignment with Jasko et al. [19], our study identifies two forms of significance motivation: individual significance driven by personal experiences and collective significance stemming from perceived group humiliation or disrespect. Our findings extend these insights by demonstrating how these forms of loss predict support for strict pandemic management policies. Our research makes a significant contribution by elucidating how personal and collective dimensions of significance loss operate distinctly to influence support for restrictive norms and extreme measures. We provide evidence that personal loss, such as financial strain and isolation, leads individuals to endorse more restrictive policies, whereas collective loss, such as underrecognition of group value (e.g., mandatory vaccines harm their belief), tends to result in a preference for less stringent measures. Although personal loss significance contributes to support for extreme measures, the findings indicate that perceived social network norms play a more substantial role in predicting such support. By applying the 3N Model, our study clarifies the mechanisms guiding individuals toward adopting social network norms that fulfill their need for significance restoration. In the context of COVID-19, perceived restrictive norms within one’s social environment appear to be a stronger predictor than internal motivational factors in explaining support for extreme measures to combat the pandemic. This insight is crucial for understanding the dynamics of the role of social environment and collective support for public health policies during the pandemic. We also build on the work of Lobato et al. [45], which explored the influence of vulnerable social environments on individuals’ susceptibility to radical narratives. Our research addresses the gap by examining how different experiences of loss guide individuals’ perceptions toward specific social network norms, influencing their support for extreme measures.

Furthermore, there is a difference in the strength of mediation. While the effect of collective loss is fully mediated by social network norm, where the direct effect is not significant, this is not the case for personal loss. Personal loss has a higher effect size as a direct effect compared to the indirect effect, even though the indirect effect is still significant. This is in line with the nature of collective loss being derived from a group [19] and, consequently, relating more to group concerns. Personal loss, on the other hand, appears to operate more directly on individuals’ motivations, with social norms playing a secondary, albeit meaningful, role. Taken together, these findings provide further support for the proposition that different types of needs—collective versus personal—can give rise to distinct effects.

These findings have significant implications for addressing public health challenges, particularly in the context of COVID-19. Our results indicate that acceptance of public health measures varies as a function of individuals’ psychological needs. By examining the differential role of psychological factors in this process, particularly with respect to compliance with restrictions in extreme conditions, we found that personal loss exerted a stronger influence than collective loss in predicting support for extreme measures such as mobility restrictions and mandatory vaccination. From a policy perspective, these results highlight the necessity of aligning public health measures with individuals’ psychological needs. For instance, policies that potentially trigger collective loss (e.g., the use of religiously proscribed substances in vaccines) may lead to resistance to mandatory vaccination. Conversely, framing vaccination as a means of reducing personal deprivation (e.g., enabling individuals to return to work) may foster greater acceptance of such measures. This approach is consistent with prior work on self-determination theory and public health communication (see Ng et al. [46]). By applying the 3N model to this issue, our study contributes to the literature by offering an alternative lens for designing interventions that promote compliance. These findings underscore the importance of considering individuals’ psychological needs in the development and implementation of public health policies.

However, this study also has several limitations. First, this limitation relates to our statistical analysis. We have not conducted a comparative analysis of country-level data. We acknowledge differences in public health infrastructure and economic conditions [47] or government policy responses [48] across countries. However, because our claims are limited to individual-level mechanisms, we chose not to include these differences in the analysis, as the effect of country differences and sampling should already be reflected indirectly in how individuals perceive and interpret prevailing social norms, narratives, and expectations in their countries. However, the lack of cross-country comparisons warrants further analysis, as it may influence perceptions of uncertainty. Good healthcare facilities may lead individuals to perceive the severity of the disease as more manageable, resulting in lower perceived uncertainty than otherwise.

Second, there is a latent variable in the model that has only two indicators. The presence of only two indicators could still result in a potential problem in estimation. We have followed the two-indicator rule [39] present in the literature, and the model converged, resulting in reliable numbers. However, future work could still benefit from employing more indicators for the latent variables.

Third, in this paper normative support for restrictive measures is implicitly assumed as the focal indicator of the narrative-network nexus in the 3N theory. Norms in 3N theory play this role, yet it must be acknowledged that in other areas of scholarship networks and narratives are often studied without norms, and vice versa, and indeed the psychological mechanisms by which narratives exert effects may be diverse [45,49,50]. More broadly, the causal interplay among 3N predictors is understudied [51], and an important direction of future research.

Lastly, the current work is a cross-sectional in design. As such, the findings should not be interpreted as causal evidence. Further studies utilizing experimental design should be conducted to clarify its causal relationships.

## Conclusion

This study analyzed cross-national data within the framework of the 3N model and demonstrated the differential roles of need deprivation—personal loss and collective loss—in shaping perceived norms that underlie support for restrictive disease-control policies. Variations in need deprivation critically influence the acceptance or rejection of collective norms. These findings highlight the importance of considering both need deprivation and prevailing collective norms when designing effective public health policies.
